# Host Cell Autophagy in Immune Response to Zoonotic Infections

**DOI:** 10.1155/2012/910525

**Published:** 2011-10-29

**Authors:** Panagiotis Skendros, Ioannis Mitroulis

**Affiliations:** First Department of Internal Medicine, Democritus University of Thrace, Alexandroupolis, Greece

## Abstract

Autophagy is a fundamental homeostatic process in which cytoplasmic targets are sequestered within double-membraned autophagosomes and subsequently delivered to lysosomes for degradation. Accumulating evidence supports the pivotal role of autophagy in host defense against intracellular pathogens implicating both innate and adaptive immunity. Many of these pathogens cause common zoonotic infections worldwide. The induction of the autophagic machinery by innate immune receptors signaling, such as TLRs, NOD1/2, and p62/SQSTM1 in antigen-presenting cells results in inhibition of survival and elimination of invading pathogens. Furthermore, Th1 cytokines induce the autophagic process, whereas autophagy also contributes to antigen processing and MHC class II presentation, linking innate to adaptive immunity. However, several pathogens have developed strategies to avoid autophagy or exploit autophagic machinery to their advantage. This paper focuses on the role of host cell autophagy in the regulation of immune response against intracellular pathogens, emphasizing on selected bacterial and protozoan zoonoses.

## 1. Introduction

The term autophagy etymologically originates from the Greek “auto”, meaning oneself, and “phagy”, meaning to eat. Macroautophagy (hereafter simply referred to as autophagy) is a dynamic biological process in which various cytoplasmic targets are sequestered within double-membraned vesicles, called autophagosomes, and subsequently delivered to lysosomes for degradation. It constitutes an evolutionarily conserved, intracellular mechanism between all eukaryotes for the maintenance of cellular homeostasis. Although, at basal, constitutive level, autophagic activity is usually low, it is markedly upregulated in response to cell stress, nutrient starvation, and immunological stimuli [1, 2]. 

Recently, substantial evidence demonstrates the pivotal role of autophagy in host defense against infections implicating both innate and adaptive immunity. In particular, the induction of the autophagic machinery in macrophages is an important innate immune mechanism resulting in inhibition of survival and direct, through degradation (xenophagy) or indirect, *via* formation and release of antimicrobial peptides, elimination of various intracellular pathogens [3]. Ligation of pathogen-associated molecular patterns (PAMPs) with pattern recognition receptors (PRRs) results in the activation of autophagy [4, 5]. Conversely, autophagy contributes to the delivery of PAMPs to endosomal PRRs indicating a bidirectional relationship between autophagy and innate immune receptors [6]. In addition, several cytokines and reactive oxygen species (ROS) that are released during the immune response to infection have been found to trigger the autophagic process [5, 7, 8]. 

Notably, autophagy contributes to antigen processing and facilitates major histocompatibility complex (MHC) class II and probably I presentation, linking innate to adaptive immune mechanisms [1, 9]. Additionally, autophagy indirectly influences the adaptive immunity against microbes by regulating the development and survival of lymphocytes [1, 9]. Finally, autophagy is an effector of Th1 immune response, which is critical for the eradication of many intracellular microbes [7].

On the other hand, several intracellular pathogens have developed diverse evasion strategies against autophagy or exploit autophagic machinery, aiming to establish an intracellular niche for long-term survival and replication [10]. Many of these pathogens are responsible for common zoonotic infections, representing an important cause of morbidity and mortality worldwide. This review summarizes the role of host cell autophagy in the regulation of immune response against intracellular pathogens, emphasizing on bacterial and protozoan zoonotic infections.

## 2. Basics of the Autophagic Molecular Pathway

The autophagic pathway is unreeled in three principal stages: initiation, elongation, and maturation. Yeast genetic studies have identified more than 30 autophagy-related genes (*ATG*), which are responsible for the triggering and regulation of autophagic machinery, although the mammalian homologs are not completely identified yet [7].

**Figure 1:**
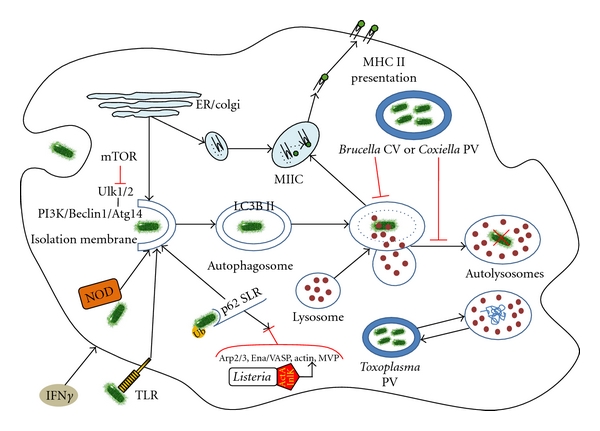
The interplay between autophagy and intracellular pathogens. Pathogen engulfment by antigen presenting cells (e.g., macrophage) and PRRs (TLR, NOD, p62) signaling induce the initiation of autophagic machinery (Ukl1/2 and PI3 K-Beclin1-Atg14 complex) and the formation of autophagosome (LC3B II), resulting in pathogen elimination by autolysosomal degradation (e.g., *Salmonella*). Th1 immune rensponse (IFN*γ*) further enhances the autophagic process. In parallel, autophagic pathway intersects the endosomal network and targets microbial antigens of phagocytosed pathogens to MHC II loading compartment (MIIC), promoting endogenous MHC II antigen presentation. Zoonotic intracellular pathogens juxtapose different mechanisms to manipulate autophagy aiming to survival and chronic parasitism, such as block (e.g., *Brucella*) or delay (e.g., *Coxiella*) of autophagolysosomal fusion, inhibition of the initiation of the autophagic machinery (e.g., cytoplasmic *Listeria*) and induction of autophagy in order to receive nutrition supplies (e.g., *Toxoplasma*). Red lines indicate negative effect. ER; endoplasmic reticulum, Ub; ubiquitin, CV; containing vacuoles, PV; parasitophorous vacuoles.

At the initiation stage, the autophagosome begins to form as isolation membrane (phagophore), originating from rough endoplasmic reticulum or probable by other membrane sources such as Golgi apparatus, mitochondria, plasma, or nuclear membrane [2, 3] (Figure 1). Atg1 (Ulk1/2 for mammals) induces this step in cooperation with a key molecular complex that constitutes class III phosphatidylinositol 3 kinase (PI3 K) hVPS34 in association with Beclin 1 (homolog of Atg6) and Atg14. Mammalian target of rapamycin (mTOR) protein kinase is thought to be the master endogenous regulator of autophagy. mTOR is coupled to Ulk1/2 complex (Ulk1/2-Atg13-FIP200-Atg101) inhibiting the induction of autophagic machinery in a nutrient-dependent manner (Figure 1). Upon nutrient/energy starvation, mTOR dissociates from the Ulk1/2 complex, which translocates at early, preautophagosomal structures exerting its inductive effect. Rapamycin is a well-characterized inhibitor of mTOR and is commonly used for the *in vitro* induction of autophagy [11]. In addition, the binding of Bcl-2 antiapoptotic protein to Beclin 1 disrupts the association of Beclin 1 with hVPS34, leading to the inhibition of autophagy [12].

 During the next step of elongation, isolation membrane enlarges and closes to form the double-membraned autophagosome that enwraps the cytoplasmic target (Figure 1). This process is regulated by two ubiquitin-like conjugation systems that are activated by Atg7, which is essential for both of them: (a) the Atg16/Atg5-Atg12 complex, which results from the Atg16 (Atg16L in mammals) in association with the Agt5-Atg12 conjugate and (b) the LC3B system which is the human homolog of yeast Atg8. Specifically, Atg16/Atg5-Atg12 complex acts as E3-like enzyme of the ubiquitin system and induces the LC3B I lipidation with phosphatidylethanolamine, resulting in LC3B II generation. LC3B-II-lipidated protein is translocated at nascent autophagosomal membrane facilitating its growth, expansion, and closure [2, 11] (Figure 1). Finally, after autophagosomal maturation to autolysosome, LC3B undergoes lysosomal degradation. Thus, LC3 II is a tracker of autophagosomes and the conversion of LC3 I to LC3 II is a widely used marker to monitor autophagic activity [13]. 

However, it has been recently suggested an Atg5/Atg7-independent alternative pathway of the mammalian autophagy that seems to be regulated by Ulk1and Beclin 1 and generates autophagosomes in a Rab9-dependent manner by the fusion of isolation membranes with vesicles derived from the trans-Golgi and late endosomes [14].

Maturation represents the final, degradative, step of autophagic molecular pathway when the autophagosomes loose the inner of the two membranes and fuse with late endosomal/lysosomal organelles, to form autolysosomes. Autolysosomes are single-membraned, acidic, vacuoles assigned to degrade sequestered material by lysosomal hydrolases [11] (Figure 1). Maturation depends on the molecular complex consisting of hVPS34—Beclin 1 in association with UVRAG (VPS38). UVRAG is a positive regulator of autophagic maturation activating Rab7 GTPase, a key element for the biogenesis and maintenance of the lysosomal compartment [15, 16]. Noteworthy, during maturation autolysosomes can also fuse with antigen processing and major histocompatibility complex (MHC) class II loading compartments, supporting MHC-II restricted endogenous antigen presentation (Figure 1) [17].

## 3. Autophagy and Immune Response to Intracellular Pathogens 

The interplay between intracellular pathogens and host immune system is critical for the development of chronic parasitism or infection clearance. Five years ago, the term ‘‘immunophagy” was introduced to cumulatively describe the contribution of autophagic machinery to all aspects of immunity. In fact, autophagy possesses regulatory and effector role influencing immune response against intracellular pathogens in many different ways [18]. The established role of autophagy as an *in vivo* defense mechanism against intracellular bacteria and protozoa has been demonstrated by studies using Atg5 knockout mice infected by *Listeria* and *Toxoplasma*, two well-characterized zoonotic pathogens [19, 20]. 

### 3.1. Antigen-Presenting Cells, PRRs, and Autophagy

Macrophages of the reticuloendothelial system are activated by autophagy (autophagic macrophage activation, APMA) in order to properly modulate intracellular microenvironment and combat the invading pathogens [10]. Autophagic elimination of intracellular microbes by APMA implicates two main ways: xenophagy and release of neoantimicrobial peptides [3, 10]. 

The best studied and well characterized is xenophagy, where microbes undergo direct degradation by autolysosomes. In contrast to nonselective or bulk autophagy that induced by nutrient deprivation or rapamycin, xenophagy involves autophagic adaptors/receptors for selective degradation of foreigner invaders [21]. This process is triggered by innate immunity receptors (PRRs), such as Toll-like receptors (TLRs) and nucleotide-binding, oligomerization-domain-(NOD-)like receptors (NLRs), following the detection of various PAMPs on cellular surface or into cytosol (Figure 1). Almost all members of the TLR family are thought to be directly or indirectly involved in the initiation and regulation of autophagic machinery against intracellular pathogens [3]. In most of these studies, the model of mycobacterial infection has been used. For example, TLR4 stimulation by lipopolysaccharide (LPS) induces autophagy in macrophages enhancing mycobacterium colocalization with the autophagosomes [22]. It seems that TLR4 signaling mediates the recruitment of Beclin-1 through dissociation of Bcl-2 inhibitor, promoting autophagy [23]. Moreover, TLR2/1 signaling regulates antibacterial autophagy pathway through functional vitamin D3 receptor activation and cathelicidin expression [24], while induction of autophagy in BCG-infected macrophages by TLR7 ligands results in pathogen elimination in a MyD88-dependent manner [25]. Of note, it is suggested that autophagy induction downstream of TLRs activation is balanced by the inhibitory effect of NF-*κ*B [26], although this matter seems to be under controversy recently [27]. 

On the other hand, autophagy acts upstream to PRRs and mediates the delivery of microbial sensors to cytosolic receptors. This process is probably related to viral infections, given that ssRNA recognition of endosomal TLR7 and production of interferon-*α* (IFN*α*) by plasmacytoid dendritic cells are suggested to be autophagy dependent [6].

Recent evidence also links bacterial sensing by cytoplasmic NLRs with the induction of autophagy. It is proposed that NOD1/2 signaling recruits Atg16L1 to plasma membrane at the sites of bacterial entry [28]. Dendritic cells from individuals with Crohn's disease that express NOD2 or Atg16L1 risk variants perform defective autophagy [29]. Interestingly, Atg16L1 polymorphism has been recently associated with an excessive production of IL-1*β* and IL-6 in humans, further indicating the implication of autophagy in the pathophysiology of Crohn's disease [30]. Together these findings support a potential role of food-borne enterobacterial infections in Crohn's disease's pathogenesis.

Sequestosome-like receptors (SLRs) represent a new group of cytoplasmic PRRs, which serve as adaptors for selective autophagy. In particular, SLRs contain an LC3 interacting region (LIR), commissioned to recognize and capture ubiquitin-coated intracellular microbes or microbes-containing compartments for xenophagy (Figure 1). SLRs contribute to xenophagy against zoonotic bacteria such as *Salmonella* and *Listeria* [2]. 

Besides the active role of xenophagy in host defense as a “microbial killer”, several lines of evidence also indicate its regulatory role as an “immune recognition enhancer” of host infected cells via the generation of antigenic microbial peptides. It is well established that autophagic pathway intersects the endosomal network and targets microbial antigens of phagocytosed pathogens to MHC II loading compartment, promoting MHC II presentation to CD4+ T-lymphocytes (Figure 1) [17]. Dendritic cells that lack key autophagy proteins such as Atg5, Atg7, or Atg16L1 are characterized by disturbances of the MHC II presentation pathway [9]. Studies of *Mycobacterium *have demonstrated that rapamycin-induced autophagy enhances MHC II presentation by mouse dendritic cells and increases antigen specific CD4+ T-cells [31]. In addition, NOD2-mediated autophagy is required for the generation of MHC II antigen-specific CD4+ T cell responses in human dendritic cells [29]. Interestingly, recent studies, albeit limited, suggest the implication of autophagic machinery to MHC I presentation of phagocytosed pathogens [32, 33]. These results support the speculation that autophagy facilitates antigenic cross-presentation process, which is critical in promoting CD8+ T-cell responses to bacteria and virus [34].

In another way of autophagic clearance, antimicrobial peptides are generated, *via* a process that implicates p62/SQSTM1 SLR. Ribosomal proteins and ubiquitin are delivered to proteolysis in autolysosomes where they are proteolytically converted into potent neoantimicrobial peptides (cryptides), further reinforcing host immune arsenal [3]. These peptides exert their antibacterial activity following the fusion of autolysosomes with parasitophorous phagosomes. Although this microbicidal mechanism has been demonstrated for *M. tuberculosis*, it is also probably related to various other intracellular bacteria like *L. monocytogenes*, *S. typhimurium* [35].

Moreover, during the APMA, phagocytosis and autophagy pathway are interconnected in a process that involves TLRs engagement and signaling. Phagocytosis constitutes a fundamental antimicrobial mechanism whereby the engulfed microbe is targeted to specialized endocytic compartments, the phagosomes, and delivered to lysosomes for degradation. Translocation of Beclin 1 and LC3 to the phagosome is related to phagosome-lysosome fusion, leading to acidification and killing of the ingested organism [36]. 

### 3.2. Cytokines and Autophagy Regulation

Several cytokines modulate autophagic mechanisms to limit intracellular pathogens replication and disturb their lifestyle. In particular, Th1 immune response is thought to be critical in host protection against intracellular pathogens, while Th2 switch has been associated with the establishment of chronicity. Of note, the principal Th1 cytokine IFN*γ* induces the autophagic control of *M. tuberculosis*, whereas Th2 cytokines (IL-4, IL-13) yield an inhibitory effect [37]. Experimental studies on macrophages have demonstrated the implication of activating immunity related GPTases (IRGs) in the autophagic clearance of different intracellular pathogens [3]. Apart from the induction of the autophagic machinery, IRGs also promote the expression of host defense proteins, such as the phagocyte oxidase, and antimicrobial peptides [38]. In mouse, various IRGs (Irgm1, Igrm3, Irga6) are directly induced by IFN*γ* (IFN-*γ*-inducible GTPases), conferring immunity to different intracellular infections within macrophages and animals [20, 39, 40]. In human, IRGM is the only IRG that has been identified since today [3]. Although it is not directly IFN*γ* inducible, it is important for the autophagic elimination of mycobacteria upon stimulation of macrophages by IFN*γ* [41]. Moreover, human genetic studies have demonstrated the association between IRGM single nucleotide polymorphisms (SNPs) and predisposition to tuberculosis, further underscoring the role of autophagy in intracellular bacterial infection [42–44]. 

TNF*α* is another Th1 cytokine, which strongly enhances the bactericidal activity of macrophages. TNF*α* has been also reported to upregulate autophagy in a ROS-dependent manner, although this effect was demonstrated in tumor cells that lack NF-*κ*B activity [26]. In addition, TNF*α* stimulates p62/SQSTM1-mediated autophagic activity and restricts the survival of *Shigella* and *Listeria* [45]. 

Furthermore, type I interferons have been implicated in antiviral autophagic response both *in vitro* and *in vivo* [46, 47]. Moreover, treatment of macrophages with interleukin IL-1 triggers the ubiquitination of Beclin 1 and the formation of autophagosomes [48].

## 4. The Crosstalk between Autophagy and Zoonotic Pathogens

Previous data strongly support the role of autophagy as an immune mechanism in the defense against intracellular pathogens. However, many pathogens successfully survive and replicate inside antigen-presenting cells (macrophages, dendritic cells) using different strategies to subvert innate immunity. Some intracellular pathogens parasitize in survival-permitting special phagosomes by remodeling the intracellular compartment to prevent phagosome maturation and phagolysosome fusion. Other intracellular microorganisms escape into the cytoplasm to avoid lysosomal degradation. A third evasion mechanism includes evasion from autophagic machinery or diversion from phagosomal to autophagic pathway and manipulation of host autophagy for microbial survival and replication [49]. 

Hereinafter, the role of host autophagy in zoonoses is described using selected examples of common bacterial and protozoan infections.

### 4.1. *Salmonella*: Autophagy Targets Enterobacterial Pathogens with Zoonotic Potential

Worldwide, foodborne diseases, and more especially diarrhoeal diseases, constitute an important cause of morbidity and mortality. *S. Typhimurium* is one of the most virulent foodborne pathogens causing gastroenteritis in humans [50].

*S. Typhimurium* invades nonphagocytic cells, such as epithelial cells and localizes within a membrane-bound compartment called Salmonella-containing vacuole (SCV) where the bacterium replicates, protected from the immune system. However, some of the SCVs are damaged and cytosolic-evaded bacteria can subsequently be targeted by ubiquitin system for autophagy [51]. Knockdown of mouse embryonic fibroblast for Atg5 was associated with increased intracellular bacterial growth, suggesting a role for autophagy in preventing bacterial escape into the cytoplasm and restricting its survival [49]. However, the *in vivo* role of xenophagy against *Salmonella* infection has been demonstrated in experimental models using autophagy defective parasites. Mutations in autophagy genes rendered these parasites susceptible to *S. Typhimurium* lethal infection, allowing intracellular survival and replication [52]. Recent studies report that the autophagic adapters p62/SQSTM1 and NDP52 are involved in the autophagic clearance of ubiquitin-coated *Salmonella* from the cytosol [53, 54]. It has been also demonstrated that phosphorylation of the autophagy receptor optineurin *via* TBK1 kinase restricts *Salmonella* intracellular growth [55].

In macrophages, *S. Typhimurium* SipB bacterial protein activates the autophagic machinery, causing autophagic cell death through the disruption of mitochondria [56]. This process might represent a host defense mechanism that destroys the bacterial intracellular cycle or, in contrast, it could be a bacterial virulence strategy [57].

### 4.2. *Listeria*: Inhibition of Autophagic Machinery Favors Survival

*L. monocytogenes *is a Gram-positive facultative anaerobe bacterium that causes febrile enteritis and invasive listeriosis, a disease that occurs primarily in newborn infants, pregnant women, elderly, and immunocompromised patients. Listeriosis is associated with a high mortality rate especially when complicated with sepsis and central nervous system involvement. The main route of acquisition of *Listeria* is the ingestion of contaminated food products such as raw meat, dairy products, vegetables, and seafood [58].

*L. monocytogenes*, in contrast to *Salmonella*, replicates in the cytoplasm after escaping from the phagosome. For this purpose, *Listeria *secretes listeriolysin O (LLO) toxin which forms pores on the phagosome membrane. Listeria has been shown to induce autophagic response in fibroblasts, epithelial cells, and macrophages in the early phase of primary infection [59, 60]. Furthermore, bacterial expression of LLO was required for autophagy induction [60, 61]. A recent study using macrophages and gene-deficient animals supports the role of IFN*γ*-inducible IRGs to cell-autonomous immunity and autophagy response to listerial infection [38]. 

However, *Listeria* has developed sophisticated mechanisms to escape from the autophagic machinery elimination. Specifically, ActA virulence protein, which is responsible for the actin-based motility of *Listeria*, has been shown to protect the bacterium from autophagic degradation. ActA promotes the recruitment of host cytosolic actin polymerization components (Arp2/3, Ena/VASP and actin) to properly mask pathogen from the recognition of ubiquitin-p62/SQSTM1-LC3 autophagic system (Figure 1), whereas ActA mutants are efficiently targeted by selective autophagy [62]. In addition, NDP52 autophagic adaptor has also been reported to target *Listeria* ActA mutant, further indicating the crucial role of ActA in resistance to the autophagic machinery [45].

Noteworthy, a second “camouflage” strategy has been recently demonstrated in *L. monocytogenes* murine infection. *Listeria* internalin InlK recruits major vault protein (MVP), a mammalian cytoplasmic protein, which disguises intracytosolic bacteria from ubiquitination and autophagic recognition promoting survival [63] (Figure 1).

### 4.3. *Brucella*: Block of (Auto)Phagolysosomal Fusion for Chronic Parasitism

Brucellosis is the commonest bacterial zoonotic infection worldwide. *Brucella* infects humans by consumption of contaminated dairy products or by occupational contact with infected animals. In humans, the disease causes high clinical morbidity and protean clinical manifestations, as any organ may be affected.* Brucella* can survive and replicate for prolonged periods within host macrophages and dendritic cells, producing chronic, and even lifelong, infections. To achieve this, *Brucella* produces various virulence factors, such as smooth LPS and outer membrane proteins/lipoproteins (Omps) that modify phagocytosis, phagolysosome fusion, antigen presentation, cytokine secretion, and apoptosis [64]. 

*Brucella* containing vacuoles (BCV) are special tight phagosomes, which represent the intracellular replication compartments. The type IV secretion system (T4SS) is a membrane-associated transporter used to deliver substrate molecules to target cells. *Brucella* T4SS is crucial for the development of the BCV in host cells as it has been described to modify the bacterial intracellular trafficking [65].

The participation of autophagy in *Brucella* spp. intracellular trafficking remains a matter of controversy [66]. In the epithelial cell line HeLa, *B. abortus* can be found in autophagosomes-like BCVs, supporting the hypothesis that pathogenic *B. abortus* exploits the autophagic machinery to establish an intracellular replication niche within the endoplasmic reticulum [67]. However, autophagic BCVs were not detected in murine macrophages [68]. Moreover, studies in cultured human peripheral blood monocytes did not demonstrate any association between BCVs and rough endoplasmic reticulum or autophagosomes, even though BCVs avoid fusion with lysosomes [69] (Figure 1).

### 4.4. *Coxiella*: The Autophagolysosomal Fusion Delays to Benefit Survival

*C. burnetii* is an obligate intracellular pathogen that causes Q fever, a worldwide zoonose with acute and chronic stages, ranging from asymptomatic to fatal disease. Farm animals and pets are the main reservoirs of infection, although a variety of species may be infected. Infection of humans usually occurs *via* inhalation of contaminated aerosols by dried placental material, fluids, and excreta of infected animals [70]. 

Once *C. burnetii* is internalized by the host cell, it is localized in early phagosomes which fuse with other vesicles to form the large parasitophorous vacuoles (PV) where this pathogen multiplies [49]. Early engagement of the autophagic machinery in the PV was associated with a delay in lysosomal fusion that enables *C. burnetii *to replicate (Figure 1). This process closely depends on T4SS virulence factor, although its effectors have not been identified yet [71, 72]. In addition, recent findings indicate that *C. burnetii* infection modulates autophagy and apoptotic pathways through Beclin 1/Bcl-2 interplay to establish a persistent infection in host cell. It seems that both PV development and the antiapoptotic effect of *C. burnetii* on host cells are affected by Beclin 1 depletion and by the expression of a Beclin 1 mutant defective in Bcl-2 binding [73].

### 4.5. *Toxoplasma* and *Leishmania*: The Autophagic Machinery against Protozoa

Toxoplasmosis is a zoonotic parasitic disease caused by the protozoan *T. gondii*. Toxoplasmosis is found in humans and in many species of animals worldwide. Cats are the primary source of infection to human hosts and fecal contamination of hands by *Toxoplasma* oocysts is a significant risk factor. Other routes of human infection include ingestion of undercooked meat and consumption of contaminated food or drink. The majority of primary infections produce no symptoms in immunocompetent persons; however, congenital toxoplasmosis may result in premature birth, hydrocephalus, chorioretinitis, deafness, or epilepsy [74].

Several lines of evidence indicate the role of autophagy in defense against *T. gondii*. In particular, CD40, a member of the TNF-receptor superfamily, signaling has been found to trigger autophagy, inducing macrophage anti-Toxoplasma gondii activity [75]. Moreover, Portillo et al. used *Toxoplasma* murine model to demonstrate the *in vivo* role of CD40-autophagic machinery for host resistance independently of IFN-*γ*. CD40 signaling upregulates Beclin 1 and triggers the elimination of *T. gondii* in microglia/macrophages by decreasing protein levels of p21, a molecule that degrades Beclin 1. These findings suggest CD40-p21-Beclin 1 as a pathway by which adaptive immunity stimulates autophagy [76]. Other animal studies also support the *in vivo* role of IFN-*γ*-inducible IRGs and ATg5 in autophagic host response against toxoplasma [20, 40]. 

Interestingly, a recent study presents evidence that Atg5-associated autophagic induction by *T. gondii* in HeLa cells and primary fibroblasts is independent of mTOR signaling, suggesting that* T. gondii* derives nutritive benefit from the upregulation of host cell autophagy to promote its intracellular growth [77]. Collectively these data probably indicate a dual role of host cell autophagy to *T. gondii *infection, acting either as a defense or as a protective mechanism (Figure 1).

*Leishmania* is an intracellular protozoan parasite that invades macrophages in the dermis after inoculation. Cutaneous leishmaniasis is the most common form of leishmaniasis, whereas visceral leishmaniasis is a severe form in which the parasites have migrated to the vital organs [78].

*L. donovani* promastigotes survive and evolve into amastigotes in phagolysosomes. Subsequently, amastigotes multiply and disseminate to the reticulo-endothelial system through vascular and lymphatic system, infiltrating the bone marrow macrophages. It is thought that the inhibition of autophagolysosome formation potentiates the survival of this parasite. Induction of autophagy by IFN*γ* or starvation increased *L. amazonensis* load and the percentages of infected macrophages from BALB/c but not from C57BL/6 mice, suggesting that autophagy may regulate the outcome of *L. amazonensis* infection in macrophages in a host strain specific manner [79]. Moreover, we have also reported the induction of the autophagic machinery during natural human bone marrow infection by *L. donovani* [78].

## 5. Conclusions

Autophagy is an important host cell defense mechanism against intracellular pathogens; many of them characterized by zoonotic potential and cause persistent/relapsing infections. The involvement of autophagy in both innate and adaptive immunity to infections is well established. On the other hand, several pathogens have evolutionary developed antiautophagic strategies or manipulate autophagic machinery for their own benefit to achieve survival and/or chronic parasitism. Further elucidation of the autophagic mechanisms implicated in the immune response or the cross talk between the immune system and pathogens is important for the discovery of biomarkers, concerning infection relapse and chronicity, as well as development of novel, autophagy-based, therapeutic approaches and vaccination strategies in livestock and humans [80].
